# Preservation of rutin nanosuspensions without the use of preservatives

**DOI:** 10.3762/bjnano.10.185

**Published:** 2019-09-19

**Authors:** Pascal L Stahr, Cornelia M Keck

**Affiliations:** 1 Department of Pharmaceutics and Biopharmaceutics, Philipps-Universität Marburg, Robert-Koch-Str. 4, 35037 Marburg, Germany

**Keywords:** nanocrystals, nanosuspension, no preservative, rutin, stability

## Abstract

Nanocrystals are used as universal approach to improve the bioactivity of poorly soluble active ingredients. They are produced by various techniques, typically yielding aqueous nanosuspensions, which are prone to microbial contamination. Preservation of nanocrystals is possible but might not always be feasible, as preservatives might interfere with other excipients in the formulations or with chemicals used in assays, cell cultures or animal models. Therefore, to enable an easier use of nanocrystals, preservative-free nanosuspensions would be a good alternative. In this study, rutin nanocrystals were frozen and stored for three months at −20 °C. The chemical, physical and microbial stability were monitored, and the results were compared to preserved nanosuspensions. The frozen nanosuspensions remained stable and possessed excellent stability over the whole time of storage, indicating that the freeze–thaw process is suitable for the production of preservative-free nanosuspensions with excellent long-term stability. The freeze–thaw process for nanosuspensions is a simple concept and is suggested as alternative, when preserved nanosuspensions cannot be used.

## Introduction

Nanocrystals for pharmaceutical use were invented in the early 1990s [1–4]. They are composed of 100% substance, are stabilized with only small amounts of surfactants, and possess particle sizes below 1 µm. According to the Ostwald–Freundlich equation nanocrystals possess a higher curvature leading to an enhanced dissolution pressure and thus to an enhanced kinetic saturation solubility [5]. Due to their small size they possess an increased surface area, resulting in an increased dissolution rate expressed by the Noyes–Whitney equation. In addition, they also possess an increased adhesiveness and thus, represent a universal, powerful and well-known formulation principle to overcome poor aqueous solubility and poor bioavailability of class-II and class-VI active ingredients of the biopharmaceutics classification system (BCS) [6–7]. Nanocrystals are already used in various pharmaceutical drug products for oral use. Examples are Rapamune^®^ (Sirolimus, Wyeth), Emend^®^ (Aprepitant, Merck), Tricor^®^ (Fenofibrate, Abbott), Megace ES^®^ (Megestrol, Par Pharm) or Triglide^®^ (First Horizon Pharmaceuticals). In 2009 the first parenteral drug product, Invega Sustenna^®^ (Paliperidone palmitate, Johnson & Johnson), was approved by the FDA. However, besides oral or parenteral administration, nanocrystals can also be used to improve the bioactivity of poorly soluble active ingredients via other routes of administration. Examples include pulmonal, ocular or dermal application [8–12].

Nanocrystals can be produced by different methods. Examples are precipitation, wet milling, high-pressure homogenization or combinations of these methods [1–4]. Regardless of the process used, all these methods will yield nanosuspensions, i.e., nanocrystals dispersed in a liquid. As liquid formulations are not always a convenient dosage form for the final drug product, in most cases nanosuspensions need to be formulated into other, more convenient, dosage forms. Depending on the route of administration this could be tablets, pellets, powders, gels or creams. However, prior to the formulation into final drug products, the aqueous nanosuspensions need to be stored, which certainly requires a sufficient stability of the nanosuspension. For this, besides chemical and physical stability, also the microbial stability needs to be considered.

One method to avoid microbial contamination of aqueous formulations during storage is the use of preservatives. In previous studies it was already found that preservatives can strongly impair the physical stability of the nanosuspensions. Reasons for this are changes of pH value or of the conductivity of the dispersion medium, or the adsorption of the preservatives onto the surface of the particles, which changes the charge of the particles (zeta potential) and forces agglomeration of the nanocrystals. To avoid instabilities of nanosuspensions only very hydrophilic and non-charged preservatives, which will not interact with the nanocrystals, should be used. Due to the above-mentioned reasons, only a few preservatives are available for the preservation of nanocrystals. Suitable preservatives for the preservation of nanocrystals include different alcohols, i.e., pentylene glycol or mixtures of phenoxyethanol and ethyl hexyl glycerol [13–15].

The limited number of preservatives and sometimes the incompatibility of these preservatives with other excipients in the final formulation are inconvenient for a successful formulation of nanosuspensions. Therefore, to enable a more convenient formulation of nanocrystals in the future, this work aimed at investigating an alternative method to maintain the microbial stability of nanosuspensions during storage.

Considering that bacterial growth strongly depends on the temperature, it was hypothesized that freezing of non-preserved nanosuspensions might prevent bacterial growth of the nanosuspensions during storage. However, the harsh conditions during freezing and thawing might also impair the physical stability of the nanocrystals and might cause agglomeration of the particles, which would then lead to a loss of the “nano properties”. Hence, in this case the method could not be exploited to preserve nanosuspensions without preservative.

**Figure 1:**
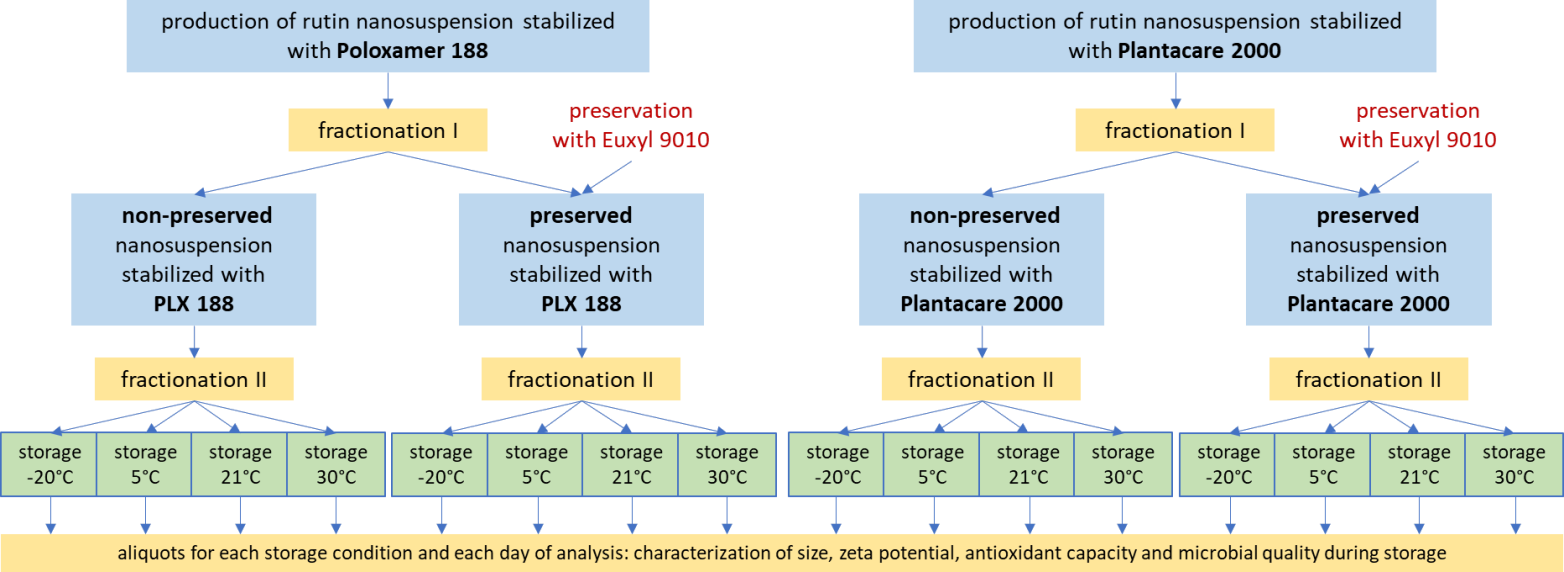
Scheme of the study. Rutin nanosuspensions with two different stabilizers were produced. The formulations were allocated, one aliquot was preserved, and the other part remained non-preserved. All formulations obtained were stored at different temperatures for three months. During this time changes in size, zeta potential, antioxidant capacity and microbial quality were monitored.

To investigate whether the freeze–thaw method is suitable for the production of long-term stable non-preserved nanosuspensions with high microbial quality, previously developed nanosuspensions containing the flavonoid rutin as model substance and either Plantacare 2000 or Poloxamer 188 (PLX 188) as stabilizers were produced by high-pressure homogenization as described previously [16–19]. Each of the nanosuspensions obtained was allocated into two parts. One part was preserved, and the other part remained non-preserved. All formulations were stored at different temperatures for a period of three months and size, zeta potential, antioxidant capacity and the microbial quality were determined and monitored over this period of time (Figure 1).

## Results and Discussion

### Production and characterization of nanosuspensions

High-pressure homogenization yielded rutin nanosuspensions with a relatively broad size distribution, i.e., polydispersity indices (PdI values) above 0.3 and some larger particles with sizes above 4 µm (Table 1). Because of this, the nanosuspensions were expected to be prone to Ostwald ripening, i.e., particle growth during storage was expected. Limited physical stability of suspensions is advantageous if a study aims at investigating different stabilizing and destabilizing effects, because in comparison to highly physically stable formulations, destabilizing effects can be detected earlier during storage, making a discrimination between stabilizing and destabilizing effects clearer.

**Table 1:** Overview of results obtained from the characterization of the nanocrystals at the day of production.

stabilizer	preservative	DLS data		zeta potential [mV]		LD data [µm]^a^	
		z-ave [nm]	PdI	in water	in medium	*d*(*v*)0.50	*d*(*v*)0.95

PLX 188	no preservative	408 ± 45	0.31 ± 0.08	−29.4 ± 2.9	−24.8 ± 2.6	1.3 ± 0.17	4.09 ± 0.15
	with preservative	412 ± 29	0.30 ± 0.05	−27.0 ± 7.8	−24.6 ± 3.0	1.3 ± 0.12	4.13 ± 0.15
Plantacare 2000	no preservative	436 ± 30	0.32 ± 0.06	−30.6 ± 4.0	−39.5 ± 2.7	1.2 ± 0.11	4.02 ± 0.11
	with preservative	447 ± 15	0.33 ± 0.06	−29.3 ± 2.7	−37.6 ± 3.3	1.2 ± 0.12	3.99 ± 0.11

^a^*d*(*v*): volumetric median diameter.

The suspension stabilized with PLX 188 yielded sizes of about 410 nm. Slightly larger nanocrystals with a slight agglomeration were obtained when Plantacare was used as stabilizer (Table 1 and Table 2). From this it was expected that non-preserved nanosuspensions stabilized with PLX 188 might possess a slightly better physical stability than the Plantacare-stabilized formulations. Upon the addition of the preservatives only very minor changes in size were observed for both formulations (Table 1) and for the Plantacare-stabilized formulation even a slight deagglomeration was determined (Table 2). Also the zeta potential values did not change significantly in both water and original dispersion medium (Table 1), again indicating no, or only a very limited, impairment of the stabilization mechanisms of the nanocrystals by the hydrophilic preservative [20].

**Table 2:** Microscopic images of the nanosuspensions stabilized with Poloxamer 188 (left) and Plantacare 2000 (right) at the day of production. Magnification: 400-fold.

	stabilized with Poloxamer 188	stabilized with Plantacare 2000

non-preserved	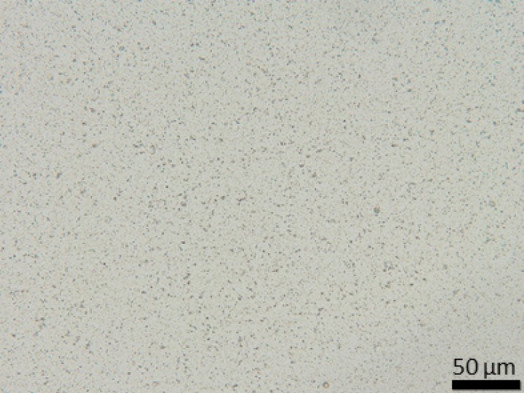	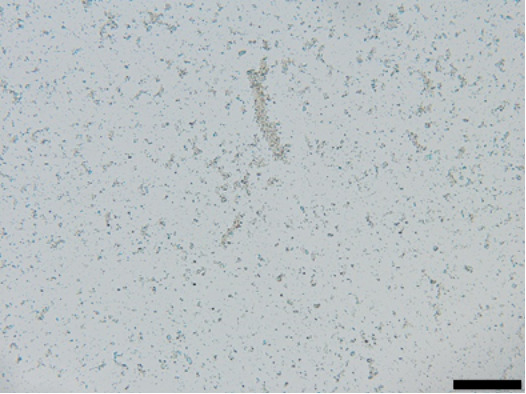
preserved	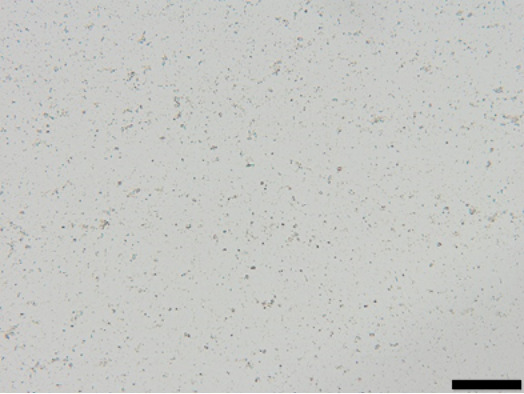	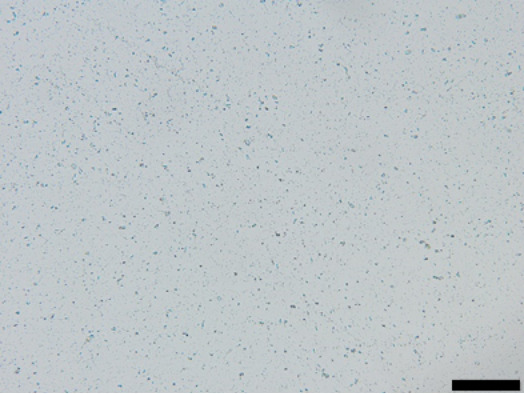

### Physical stability

**Figure 2:**
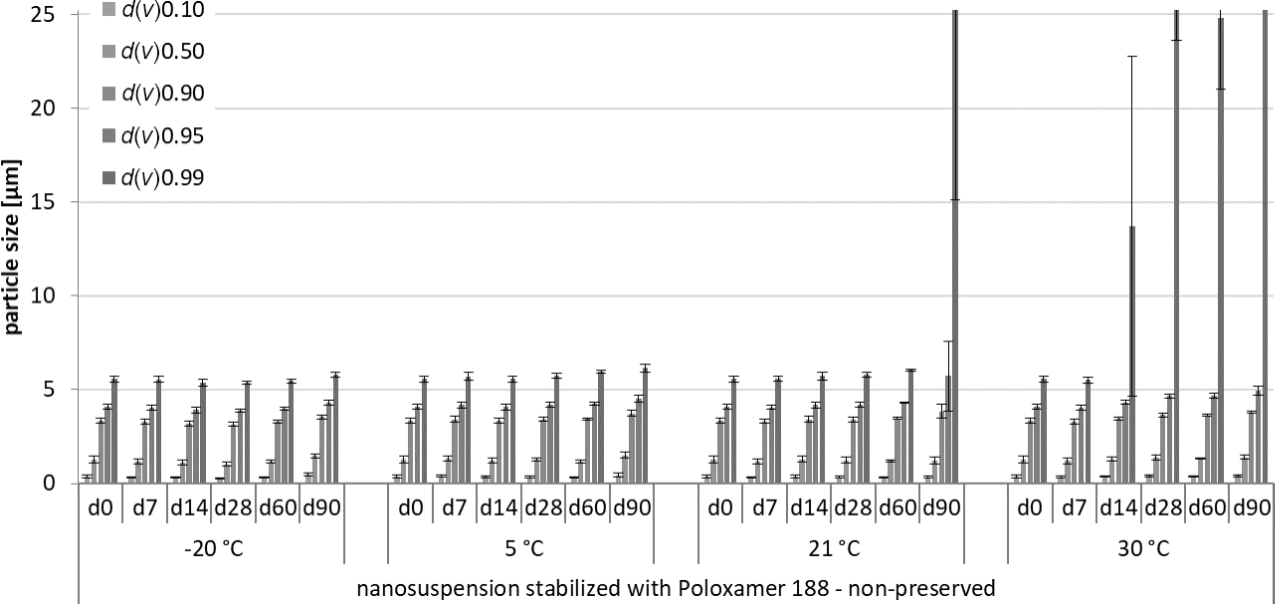
Physical stability during three months of storage at different storage temperatures for the non-preserved nanosuspensions stabilized with Poloxamer 188 (LD data).

**Figure 3:**
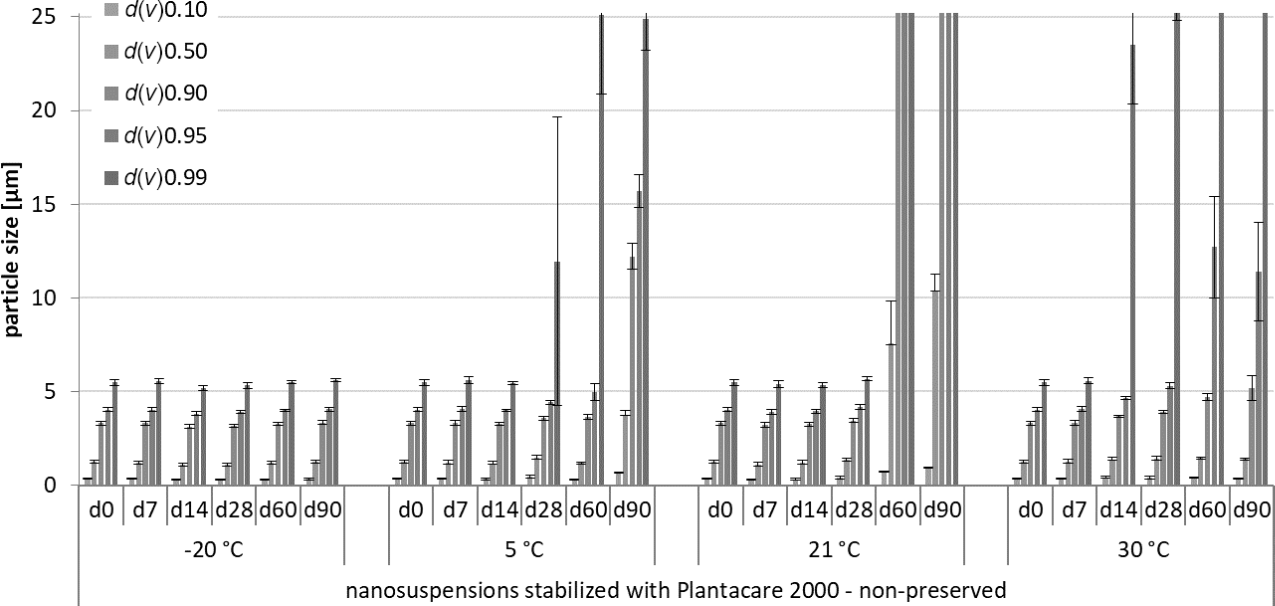
Physical stability during three months of storage at different storage temperatures for the non-preserved nanosuspensions stabilized with Plantacare 2000 (LD data).

**Figure 4:**
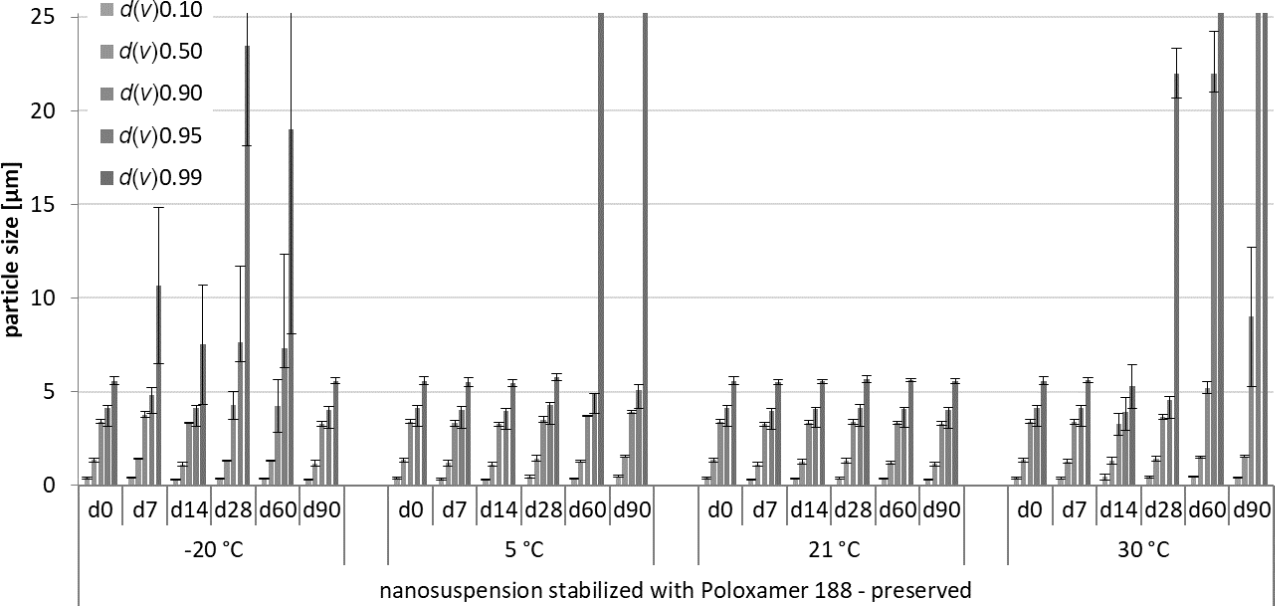
Physical stability during three months of storage at different storage temperatures for the preserved nanosuspensions stabilized with Poloxamer 188 (LD data).

**Figure 5:**
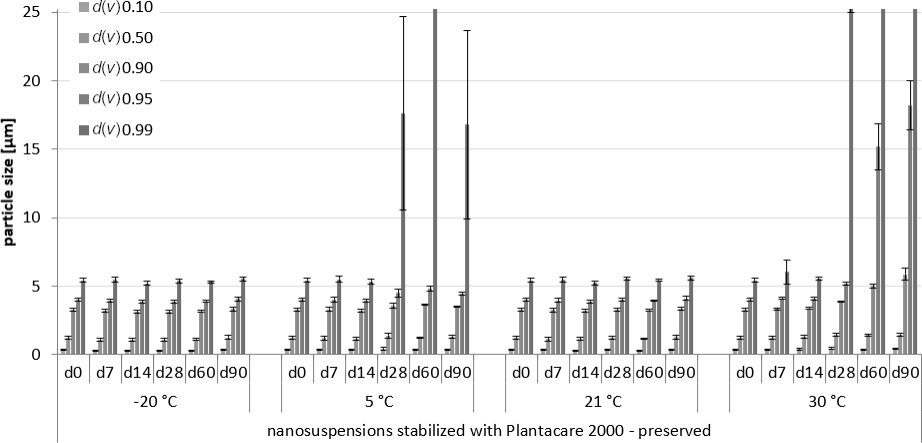
Physical stability during three months of storage at different storage temperatures for the preserved nanosuspensions stabilized with Plantacare 2000 (LD data).

**Figure 6:**
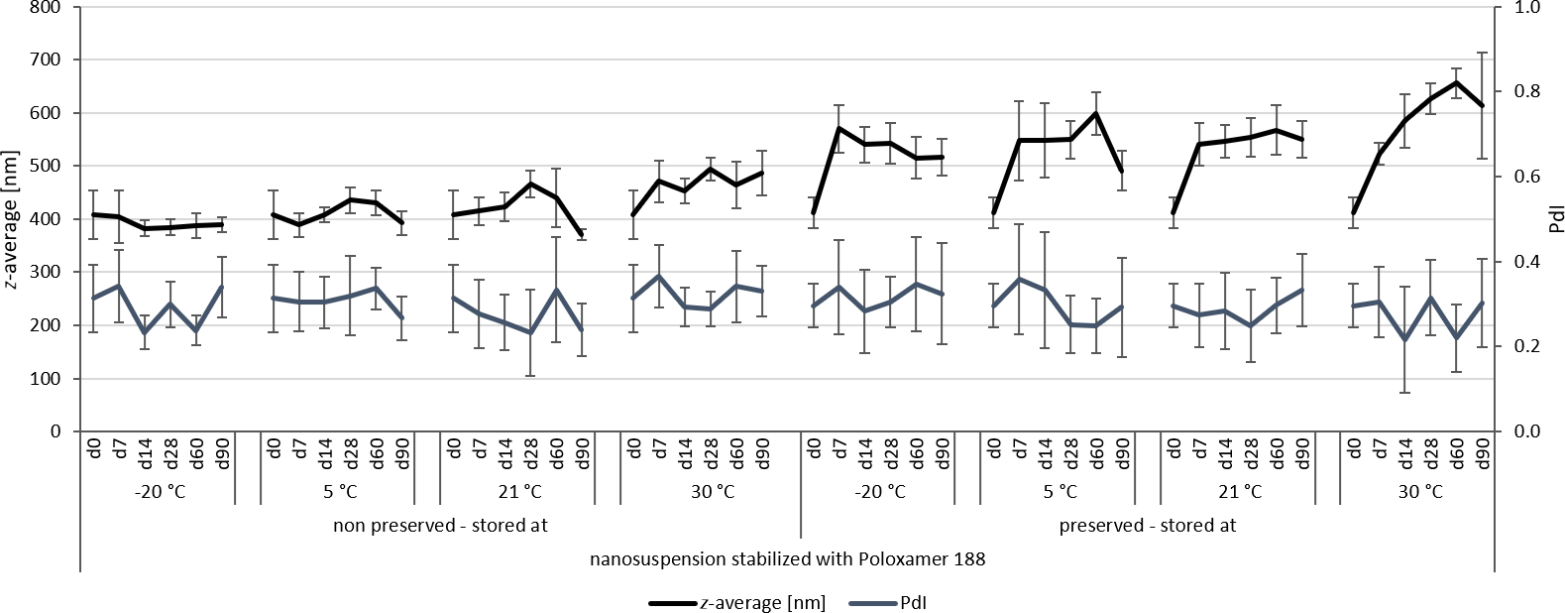
Physical stability during three months of storage at different storage temperatures for the nanosuspensions stabilized with Poloxamer 188 (DLS data).

**Figure 7:**
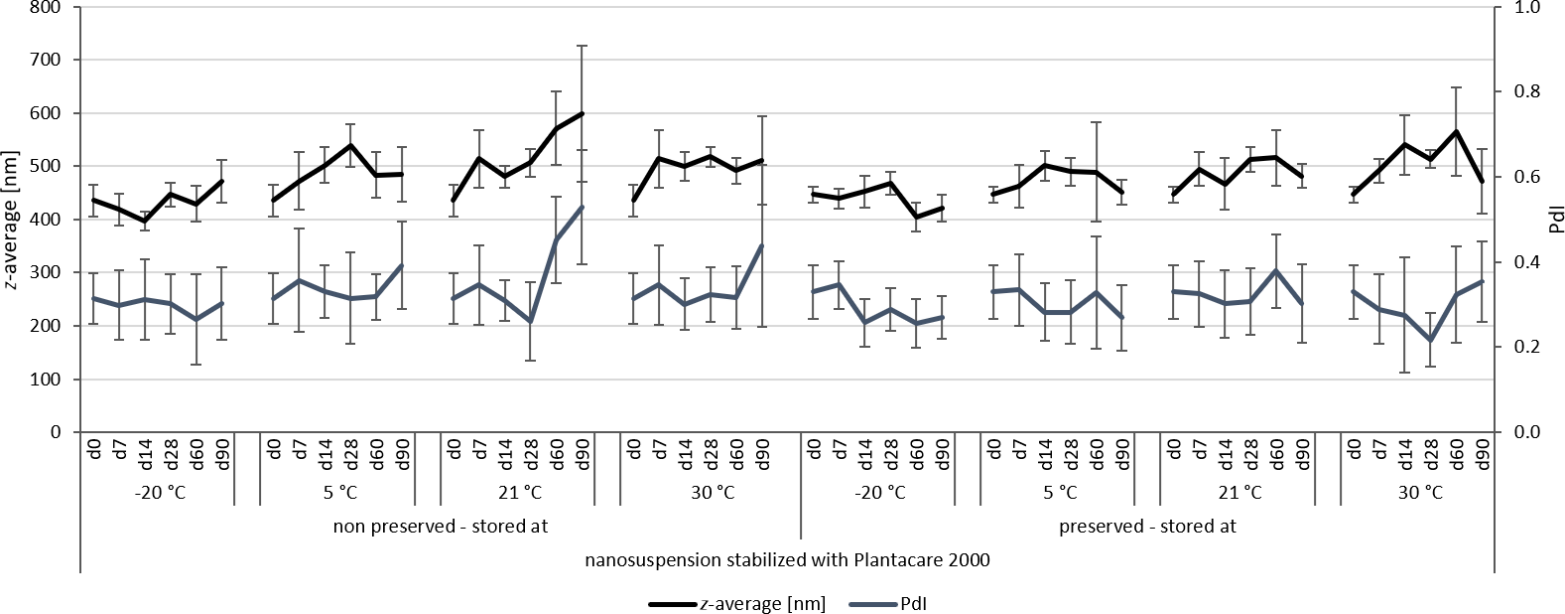
Physical stability during three months of storage at different storage temperatures for the nanosuspensions stabilized with Plantacare 2000 (DLS data).

The physical stability was assessed by size measurements over a period of three months of samples under all storage conditions. Increases in *d*(*v*) values, *z*-average, and polydispersity index (PdI) over time indicated instability. The laser diffractometry (LD) data and the dynamic light scattering (DLS) data obtained from this part of the study are shown in Figures 2–7.

**Figure 8:**
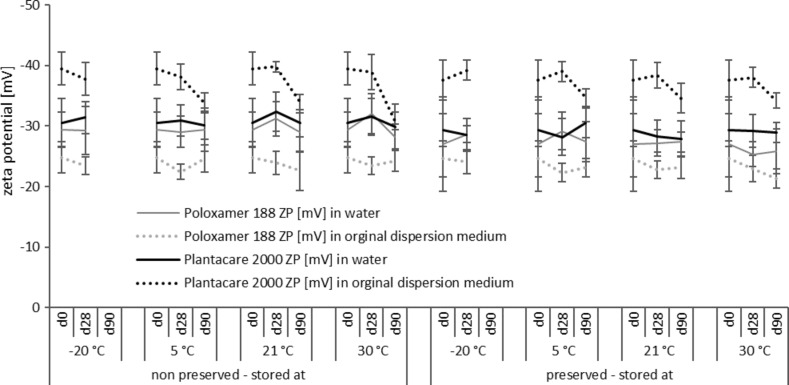
Determination of zeta potentials of the non-preserved and preserved rutin nanosuspensions during three months of storage at different storage temperatures.

For the non-preserved nanosuspensions, increasing storage temperatures reduced the physical stability of the non-preserved suspensions (Figure 2, Figure 3, Figure 6, Figure 7). Consequently, the least stable formulations were obtained when the formulations were stored at 30 °C. Reasons for this are the more pronounced particle growth due to Ostwald ripening at elevated temperatures and/or destabilization due to accelerated bacterial growth, which might excrete compounds that contribute to changes in pH value or conductivity in the medium or interact with the particles. Such changes should become visible in the zeta potential values. However, this was not the case in this study (Figure 8). Therefore, the observed destabilization at elevated temperatures of the non-preserved nanosuspensions might be more related to Ostwald ripening. The assumption is also underlined by the fact that the Poloxamer-stabilized formulations, which possessed a narrower size distribution, i.e., no agglomerates at the day of production (c.f. Table 2), were found to be more stable than the Plantacare-stabilized formulations with a broader size distribution due to a slight agglomeration of the nanocrystals at the day of production.

The trend for physical stability was different for the preserved nanosuspensions. The most stable formulations were obtained when the samples were stored at room temperature. Lower (except freezing) and higher temperatures reduced the stability (Figures 4–7). Reasons for this cannot be explained completely but might be due to the presence of the preservatives that were added to the samples at room temperature. Changes in temperature might change the interaction between preservative and particles and thus the stability. More research is needed to understand these phenomena in detail.

Most interesting results were obtained for the samples that were stored in frozen state. Physically stable formulations, i.e., without pronounced changes in *z*-average, PdI and LD values, were obtained for the non-preserved suspensions when stored at −20 °C. These results were not expected, because in general it is assumed that freezing of colloidal formulations leads to changes in the stabilizing layer and the particle interactions, being the cause for agglomeration of the nanoparticles. Especially freezing of nanosuspensions that contain dissolved active ingredients in “supersaturation” is hazardous, because due to the reduction in temperature, re-crystallization of dissolved active ingredients can easily occur. Nevertheless, the data obtained from this study indicate that it is possible to freeze–thaw nanosuspensions with good steric and/or electrostatic stabilization, without impairing their physical stability. The later fact seems to be highly important, especially when looking at the data obtained for the frozen and preserved nanosuspensions. Here, it was found that preserved nanosuspensions that were stabilized with PLX 188 became unstable during the freeze–thaw process, whereas Plantacare-stabilized formulations remained stable.

Reasons for the differences might be the different stabilizers that interact differently with the preservative. Poloxamer 188 is a non-ionic surfactant, providing steric stabilization for the nanocrystals. In general, a thick surfactant layer that is indicated by a zeta potential (ZP) near zero should be obtained for good steric stabilization [21]. However, in our study ZP analysis revealed that the thickness of the layer is relatively low (ZP > 20 mV, cf. Table 1). Hence, steric stabilization of the formulation stabilized with PLX 188 is relatively poor. Upon the addition of the preservative to the formulations stabilized with PLX 188 a very limited decrease in ZP was detected when the suspensions were analysed in original dispersion medium (c.f. Table 1). This might indicate that small amounts of the non-charged preservative are adsorbed onto the surface of the nanocrystals where it might interact with the polymer layer. This interaction might cause a re-arrangement of the already thin stabilizing layer around the nanocrystals and might therefore explain the decreased stabilization efficacy of the poloxamer in the preserved formulations. The destabilizing effect of PLX 188 in combination with other excipients in nanocrystal formulations was also shown by a study of Beirowski and co-workers, who showed that some combinations of poloxamer and cryoprotectant were unsuitable for stabilizing nanocrystals during a freezing process [22].

In contrast to PLX 188, Plantacare, which is an alkyl polyglucoside, is mostly providing electrostatic stabilization. This can be seen by the differences in ZP analysed in water and original dispersion medium, respectively. The zeta potential is about −40 mV in original dispersion medium and is reduced to about −30 mV when analysed in water, because upon dilution with water surfactant is “washed off” from the particle surface, which results in less electrostatic stabilization of the particles. However, due to its chemical structure, Plantacare is also able to provide steric stabilization. This leads to an excellent stabilization capacity combining steric and electrostatic stabilization [23–24]. Upon addition of the preservative only very small differences in ZP values were detected for both, water and original dispersion medium (c.f. Table 1), indicating only very minor impairment of the preservative. In fact, Plantacare provides a very efficient stabilization mechanism, which is not significantly impaired by the addition of the preservative. This explains the slightly better physical stability than that of the preserved PLX-stabilized nanosuspensions.

### Antioxidant capacity

**Figure 9:**
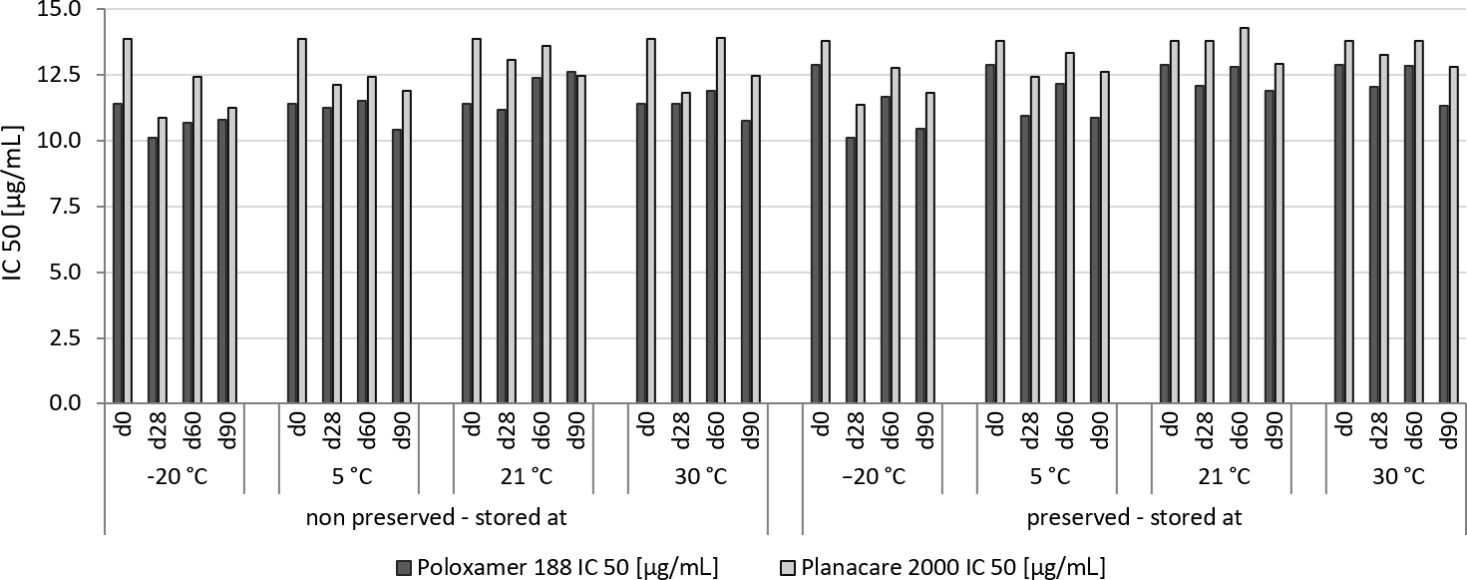
Antioxidant capacity, determined as IC_50_ value, of the rutin nanosuspensions during storage. No changes in IC_50_ values were determined during three months of storage, thus indicating good chemical stability of all rutin nanosuspensions.

The antioxidant capacity (AOC) was measured with the DPPH assay in which the IC_50_ value is determined. The IC_50_ value determines the amount of antioxidant needed to scavenge 50% of the free radical. Consequently, the smaller the IC_50_ value the higher is the antioxidant capacity. In this study, the IC_50_ values for the different formulations did not change during storage, independent on preservative, storage time and storage temperature (Figure 9). Hence, all these parameters did not affect the AOC of the formulations. As the AOC is an indirect measure for the chemical stability, data indicate excellent chemical stability of all aliquots during storage. The data are in good agreement with a recent study by Müller et al. in which the authors could prove chemical stability of a rutin nanosuspension for more than nine years [25].

### Microbial quality

**Figure 10:**
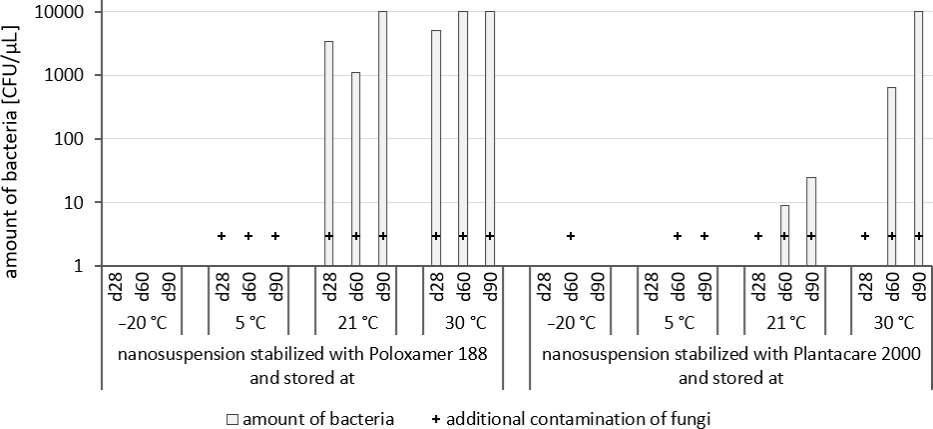
Determination of microbial activity (CFU) during three months of storage.

The growth of bacteria and fungi was determined for all formulations after one, two and three months of storage. All preserved formulations showed excellent microbial quality. No fungi or bacteria were detected during the three months of storage (Figure 10). For the non-preserved nanosuspensions, data indicated that for all formulations the number of bacteria was fairly low upon the production with high-pressure homogenization, which is a well-described technique to reduce the number of bacteria in liquids [26]. The growth of microorganisms during storage was temperature-dependent and was also found to be slightly influenced by the type of stabilizer, i.e., a slightly lower and slower increase in microbial growth was found for the Plantacare-stabilized formulations (Figure 10 and Table 3). A possible reason for this observation could be the antimicrobial activity of the stabilizer Plantacare, which was already described in previous works by Jurado and co-workers [27–28].

**Table 3:** Determination of microbial quality during three months of storage. P188: rutin nanosuspension stabilized with Poloxamer 188, PLC: rutin nanosuspension stabilized with Plantacare 2000, (+) = preserved with Euxyl 9010, (−) non-preserved.

	storage temperature			
	−20 °C	5 °C	21 °C	30 °C

d28 bacteria	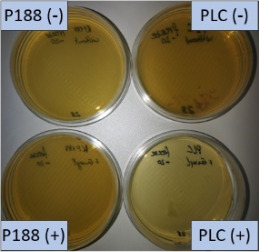	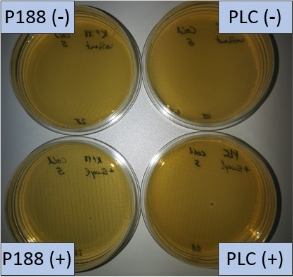	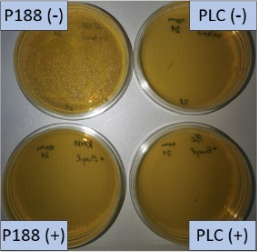	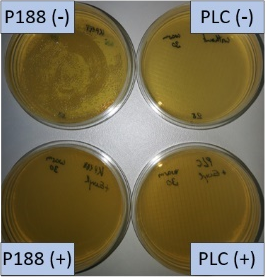
d28 fungi	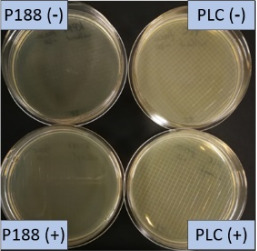	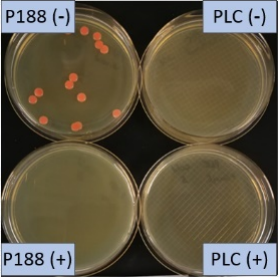	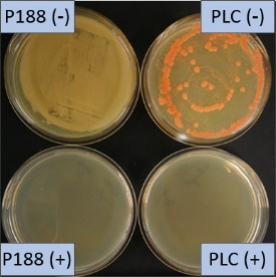	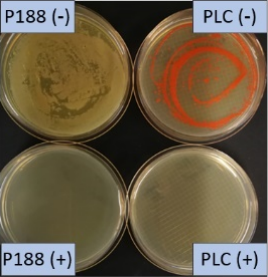
d60 bacteria	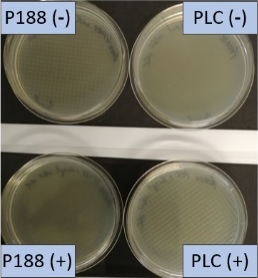	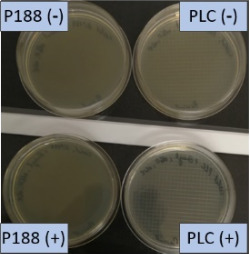	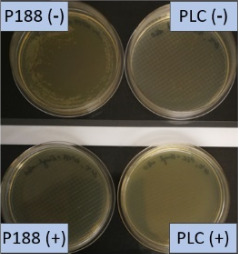	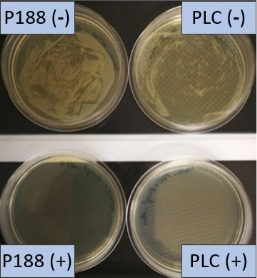
d60 fungi	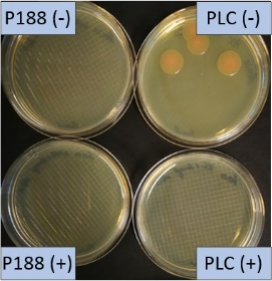	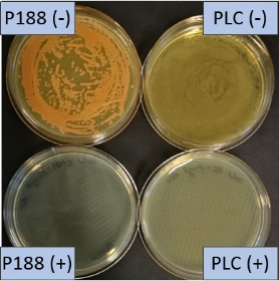	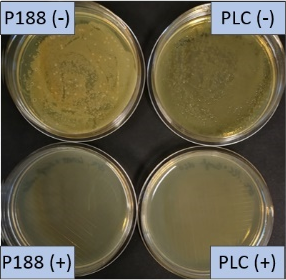	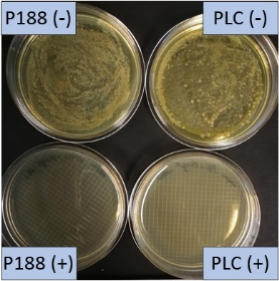
d90 bacteria	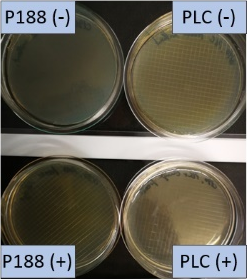	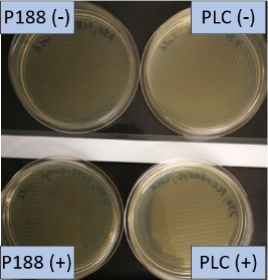	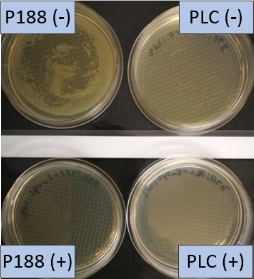	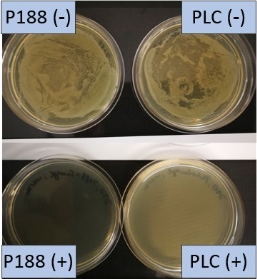
d90 fungi	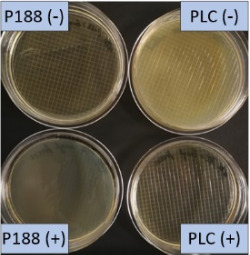	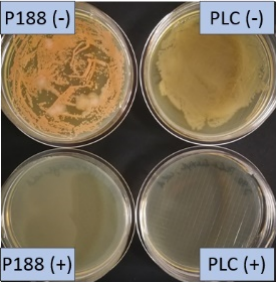	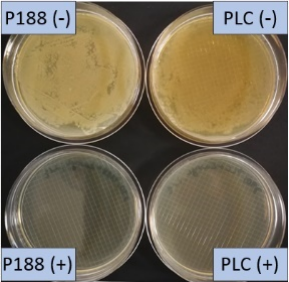	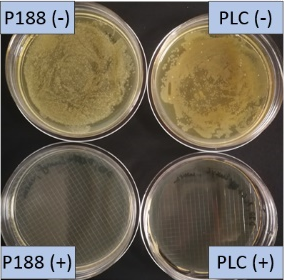

Finally, it was found that for all formulations that were stored in frozen state at −20 °C no bacterial growth occurred. Hence, the hypothesis, that storing nanosuspensions after production in frozen state might prevent bacterial growth during storage without the use of preservatives, could be confirmed by this set of data. The same trend was also observed for the growth of fungi. However, there was one exception, which was observed for the Plantacare-stabilized formulation at day 60 of storage. At this time point a slight contamination with fungi was observed for one aliquot (Figure 10 and Table 3). However, no contamination with fungi was observed at the next time point, i.e., after 90 days of storage. In fact, after three months of storage at −20 °C and upon thawing all non-preserved nanosuspensions were found to possess an excellent microbial quality, as no fungal and no bacterial growth was detected.

These findings so far are very promising and could enable a new concept to produce preservative-free nanosuspensions that can be stored over a longer period until further use or processing into final dosage forms. Preservative-free aqueous nanosuspensions would be a convenient formulation principle, because there will be no need to take possible interactions with preservatives and/or other excipients into consideration. Allergies of consumers and/or regulatory hurdles can also be circumvented with this concept.

The freeze–thaw concept is simple and can be exploited not only in industry but also in early drug development, where nanosuspensions are often used for early formulation of poorly soluble drug candidates. In this environment, the freeze–thaw concept could improve the predictability of screenings. At present, due to the lack of microbial stability, nanosuspensions need to be prepared shortly before the experiments, i.e., assays, cell culture or in vivo studies, are performed. Any repeating of the tests or continued tests will require the production of new suspensions, which might possess slightly different properties, which in turn might then cause differences in the (in vivo) data. By using thawed nanosuspensions from only one batch, these variations could be circumvented.

In conclusion, the freeze–thaw concept was shown to be a simple method to prevent microbial contamination during storage of aqueous rutin nanosuspensions. The new method is believed to enable new possibilities for the use of nanosuspensions and thus can be seen as a highly promising concept, not only in pharma, but also in food and cosmetics. Next steps should now investigate if the concept can also be exploited for other active ingredients and other stabilizers.

## Conclusion

Preserved and non-preserved rutin nanosuspensions stabilized with different stabilizers were produced in this study and were stored for three months at different storage temperatures. During this time physical stability and microbial quality were monitored. In addition, the antioxidant capacity, as an indicator for the chemical stability, was assessed. All formulations were chemically stable over the whole time of observation. Physical stability was influenced by the type of surfactant, the preservative and the storage temperature. Preserved samples were only stable when stored at room temperature. Storage at higher or lower temperatures strongly impaired their physical stability. However, the microbial quality was excellent for all preserved nanosuspensions. Non-preserved samples possessed a better physical stability than the preserved nanosuspension. Proving again that preservatives impair the physical stability of nanosuspensions. Most interestingly, it was found that freezing did not alter the physical stability of the non-preserved suspensions. Hence, nanosuspensions could be frozen, stored up to three months at −20 °C and possessed unchanged particle sizes upon thawing. Storage at −20 °C also prevented bacterial growth of the non-preserved nanosuspensions, whereas storage at higher temperatures caused microbial contamination of the suspensions. The freeze–thaw concept was therefore found to be a suitable method to produce not only physically and chemically but also microbially stable rutin nanosuspensions. More research is now needed to investigate if the method can also be transferred to other nanosuspensions or nanosized formulations. All in all, the method seems to be a promising method to enable long time storage of aqueous nanosuspensions with excellent stability and without the use of preservatives. It can be used for improved formulation development of poorly soluble active ingredients in both lab scale and industrial scale.

## Experimental

### Materials

Rutin was purchased from Denk Ingredients GmbH (Germany). The stabilizers Poloxamer 188 (PLX 188, Kolliphor^®^ P 188) and alkyl polyglucoside C8-C16 (Plantacare^®^ 2000 UP) were kindly provided from BASF AG (Germany). The preservative was composed of 90% (w/w) 2-phenoxyethanol and 10% (w/w) of 1,2-propanediol as ready to use mixture (Euxyl^®^ 9010) and was obtained from Schülke & Mayr GmbH (Germany). Purified water was obtained from a PURELAB Flex 2 (ELGA LabWater & Veolia, Germany). All other analytical chemicals were of analytical grade and used as received.

### Methods

#### Production of nanosuspensions

Rutin nanosuspensions [16–19] were produced by high-pressure homogenization (HPH) using an EmulsiFlex-C50 (Avestin, Germany). For this, bulk suspensions containing 5% (w/w) rutin and 1% (w/w) surfactant were prepared. The pre-dispersions were homogenized with a high-speed stirrer (D-27, Miccra GmbH, Germany) at 24,000 rpm for 5 min in continuous mode and were subsequently subjected to HPH (20 cycles at 1500 bar). During homogenization and between each cycle the suspensions were cooled to below 10 °C by using a cooling bath to avoid heating of the suspensions and subsequent agglomeration of the crystals [29].

#### Characterization of nanosuspensions

**Determination of particle size and physical stability:** Nanosuspensions were characterized regarding size by three different and independent methods. The hydrodynamic diameter and the polydispersity (*z*-average (*z*-ave) and PdI) were analysed by dynamic light scattering (DLS) with a Zetasizer Nano ZS (Malvern Panalytical GmbH, Germany). As DLS measurements, when used as stand-alone method for the characterization of submicron-sized particles, can be misleading because larger sized particles are not detected [30–33], light microscopy (Olympus BX53, equipped with an Olympus SC50 CMOS color camera, Japan) and laser diffraction (LD) were used as additional techniques to securely detect possible larger particles and agglomerates within the suspensions. By LD analysis the volumetric median diameters *d*(*v*)0.1, *d*(*v*)0.5, *d*(*v*)0.9, *d*(*v*)0.95 and *d*(*v*)0.99 were analysed with a Mastersizer 3000 (Malvern Panalytical GmbH, Germany). Particle diameters were calculated with Mie-theory by using 1.57 as real refractive index and 0.01 as imaginary refractive index.

The zeta potential (ZP) of the nanocrystals was determined in water (adjusted to a constant conductivity of 50 µS/cm) and in original dispersion media, i.e., surfactant solution, containing either 1% (w/w) PLX 188 or Plantacare 2000, respectively. Measurements were performed via laser-Doppler-anemometry (LDA) by using a Zetasizer Nano ZS (Malvern Panalytical GmbH, Germany), which determines the electrophoretic mobility (EM), which was then converted into the ZP by using the Helmholtz–Smoluchowski equation [21].

**Determination of antioxidant capacity:** The antioxidant capacity was assessed by calculating the IC_50_ value, which was determined by using the DPPH assay [34]. DPPH (1,1-diphenyl-2-picryl-hydrazyl, Sigma-Aldrich, Germany) is a free radical that can be reduced by antioxidants. Upon reduction the colour of the free radical changes and thus the amount of reduced DPPH can be accessed via UV–vis spectroscopy. For the determination of the IC_50_ values, 100 µL of the samples containing different concentrations of the nanocrystals (200, 100, 50, 25, 12.5, 6.25, 3.125 µg/mL) were added to 100 µL of a 0.3 mM methanolic solution of DPPH. After 30 min incubation time in the dark, the absorbance was measured by a UV–vis plate reader (Multiskan GO, Thermo scientific, Germany) at 517 nm. The inhibition activity (inhibition [%]) was calculated as


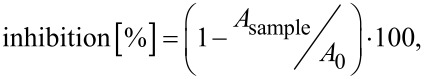


where *A*_sample_ is the absorbance of the sample and *A*_0_ is the absorbance of the control (DPPH solution). The resulting linear function of inhibition against concentration was used to calculate the IC_50_ value (µg/mL). The IC_50_ value represents the concentration needed to scavenge 50% of the free radical. Rutin, which was used in this study as model drug, is a well-known antioxidant. Hence, if chemical degradation of the active ingredient occurs, changes in the IC_50_ value during storage can be observed [35]. As methanol is a good solvent for rutin, the addition of the nanocrystals to the methanolic DPPH solution led to a complete dissolution of the rutin nanocrystals. Hence, all rutin remaining in the formulations was dissolved during the test and thus the DPPH assay was used as a surrogate for the determination of the chemical stability.

**Determination of microbial quality:** To verify the biological stability, a simple agar plate test after Ph. Eur. 8 was used. The agar (Müller–Hinton agar, Sigma) was dispersed in water, autoclaved and directly poured into sterile petri dishes (60 mm in diameter) at 28, 60 and 90 days after preparation of the nanosuspensions and used immediately after cooling. Subsequently, 10 μL of the nanosuspension or a 1:100 dilution in water were distributed evenly over the entire surface by means of a cell spreader. For detection of existing bacteria, the agar plate containing the suspension or dilution was incubated for 24 h at 36 °C and 90% humidity. Fungal contamination was detected after 7 days at 25 °C storage by using undiluted nanosuspensions. For evaluation, the visible bacterial colonies were counted, or the presence of fungal growth was noted. With dense colonization, the number of colony forming units (CFU) was set to 10000 CFU/μL.
